# Anastasis: Return Journey from Cell Death

**DOI:** 10.3390/cancers13153671

**Published:** 2021-07-22

**Authors:** Victoria Zaitceva, Gelina S. Kopeina, Boris Zhivotovsky

**Affiliations:** 1 Faculty of Medicine, MV Lomonosov Moscow State University, 119991 Moscow, Russia; zaytseva-victoria-pharm14-15@mail.ru (V.Z.); lirroster@gmail.com (G.S.K.); 2 Division of Toxicology, Institute of Environmental Medicine, Karolinska Institutet, Box 210, 17177 Stockholm, Sweden

**Keywords:** anastasis, apoptosis, mitochondria, stress, survival

## Abstract

**Simple Summary:**

Despite the development of new anticancer therapies, resistance and recurrence after treatment remain one of the greatest challenges in modern oncology. Anastasis—a recently described phenomenon—could be one explanation for why cytotoxic drugs fail to kill cancer cells. We aim to review current experimental data on cell death reversal and to discuss the possible mechanisms behind anastasis.

**Abstract:**

For over 20 years, it has been a dogma that once the integrity of mitochondria is disrupted and proapoptotic proteins that are normally located in the intermembrane space of mitochondria appeared in the cytoplasm, the process of cell death becomes inevitable. However, it has been recently shown that upon removal of the death signal, even at the stage of disturbance in the mitochondria, cells can recover and continue to grow. This phenomenon was named anastasis. Here, we will critically discuss the present knowledge concerning the mechanisms of cell death reversal, or development of anastasis, methods for its detection, and what role signaling from different intracellular compartments plays in anastasis stimulation.

## 1. Introduction

Cells constantly fight with external stress and can eliminate any subsequent damage. As a result of successful repair, the cell life cycle continues. In the case of damage that is too severe, the cell commits suicide to avoid further tissue and organ destruction, and to eradicate genetically unstable and dangerous cells. However, if the cell death mechanism does not operate properly, «unwanted» cells can go down the road of malignancy. The main goal of anti-cancer treatment is the elimination of malignant cells via the induction of cell death [1]. Importantly, resistance to cell death is one of the essential hallmarks of cancer, which permits cells to multiply in an uncontrolled manner and continue the life cycle with accumulated damage and genetic instability [2]. During the past decades, several mechanisms by which cancer cells can avoid cell death were described. Among them are the overexpression of antiapoptotic Bcl-2 family proteins and the inactivation of p53 [3]. Based on this, a number of successful treatment approaches have been developed; unfortunately, almost all have faced the same shortcomings—the creation of acquired drug resistance. The efficacy of anticancer therapy varies depending on the type and stage of the tumor, drug combination and dosage, and many other factors, but the therapy rarely kills all the cancer cells. There are examples of positive selection in cancer cells that acquire mutations that allow them to resist therapy. The riddle is why some cells in a clonal population survive when facing a death-inducing stimulus while their clonemates die [4]. Another angle to investigate is whether these surviving cells are truly alive, or whether they have returned from the «dead population». In that light, the recently described phenomenon named anastasis is interesting to discuss. Anastasis implies that cells in the process of committing death in certain cases, after the stimulus is gone, stop the cell suicide process and return to life [5]. Anastasis is different from the other two ‘if not death’ cell fates after treatment—senescence and mitotic catastrophe—as cells retain their proliferative capacity and even enhance it, which make anastasis an undesirable event during anti-cancer therapy. Below, we aim to expand limited knowledge about anastasis by discussing the possible mechanisms by which cells can reverse death, which was thought to be an irreversible process once it had reached a late stage.

## 2. A Few Historical Remarks

The first indications that apoptosis, the best-studied form of programmed cell death, can be reversible date back to the end of the 20th century. It was shown that apoptosis might not represent death in heart failure, but rather the programmed cell survival of cardiomyocytes and the likelihood of reverse remodeling. In this case, although many cardiomyocytes display such hallmarks of apoptosis as cytochrome *c* release and caspase-3 activation, they surprisingly demonstrate normal nuclei morphology and remain unaffected by the apoptotic process [6]. These findings for the first time point to a possible cessation of the apoptotic process before it is completed. Importantly, several groups used histochemical and ultrastructural approaches to support these biochemical data [7]. This phenomenon of a lack of terminal morphological features of apoptosis despite the activation of the apoptotic pathway was named by the authors as «apoptosis interruptus» [8] and suggested to have important pathogenetic and therapeutic implications for heart failure.

Using a single-cell analysis of TRAIL (TNF-related apoptosis-inducing ligand)-treated cells, it was found that a regulatory link between initiator and executor caspases can be impaired, creating a physiologically unknown state of partial cell death with the potential to generate genomic instability [9]. Moreover, it has been shown that TRAIL, in addition to activating receptor-mediated cell death, can induce DNA damage in surviving cells, an effect that is also dependent on caspase-8-mediated activation of caspase-activated endonuclease (CAD). After exposure to sub-lethal concentrations of TRAIL, surviving cells showed high mutation rates [10]. Importantly, not only treatment with TRAIL, but also treatment with sublethal doses of various chemical agents led to cell survival after caspase activation [11]. Ionizing radiation of breast cancer cells revealed their survival, even in the presence of activation of the main executor caspase-3. All of these observations were called «failed apoptosis» [12]. Finally, in 2012, Tang and colleagues introduced the term anastasis, describing the survival of HeLa cells upon the washing out of ethanol after treatment [5]. Notably, the general biological significance of anastasis has been detected not only in various in vitro cell models but also in an in vivo model of Drosophila melanogaster development [13].

## 3. A Mitochondria-Anastasis Link

Apoptosis is triggered by a wide spectrum of stimuli, which all might activate two main pathways: the extrinsic (the death receptor-mediated) and the intrinsic (the mitochondria-mediated) pathways. The death receptor pathway is initiated by the binding of the death-inducing ligand with a death receptor on the cell surface. The mitochondrial pathway is initiated by diverse intrinsic signals such as DNA damage, oxidative stress, and growth factor deprivation, which eventually converge at the level of the mitochondria. This intracellular compartment fulfills an important function in apoptosis regulation, both in signaling and execution, and might act as a switchboard between various cell death modalities. Thus, mitochondria outer membrane permeabilization (MOMP) plays a pivotal role in the regulation of the mitochondria-mediated apoptotic pathway, leading to the release of several pro-apoptotic proteins from the intermembrane space of mitochondria. Among them is cytochrome *c*, which being in cytosol binds to APAF-1 (apoptotic protease activating factor 1), and in the presence of dATP recruits procaspase-9, leading to the formation and activation of the apoptosome complex. Active within the apoptosome, caspase-9 cleaves and activates the executioner caspases-3, -6, and -7, which mediate downstream events such as cell shrinkage, chromatin condensation, nuclear fragmentation, and the formation of apoptotic bodies [14,15]. As a key step, mitochondrial permeabilization is strictly regulated by interactions between Bcl-2 family proteins. This family consists of three groups of structurally related proteins: pro-survival Bcl-2 proteins (Bcl-2, Bcl-XL, Mcl-1, etc.), pro-apoptotic pore formers (Bax, Bak), and pro-apoptotic BH3-only proteins (Bim, Puma, Bid, Noxa, etc.) [16]. The ratio of cell survival and cell loss is determined by the balance between these pro-survival and pro-death proteins. Apoptosis is triggered when this balance is shifted towards the latter and, thereby, controls cell number homeostasis.

As mentioned above, for a long time it was a dogma that once the mitochondrial integrity is disrupted, cell death becomes inevitable. However, some types of cells can survive and recover after the removal of MOMP-inducing stimuli [11]. The ethanol-treated HeLa cells exhibited the hallmarks of apoptosis (including cytochrome *c* release, caspase-3 activation, and DNA fragmentation) [5]. Ethanol is thought to activate the intrinsic apoptosis pathway which most likely occurs with the formation of MOMP [17]. After washing out the ethanol, the vast majority of cells surprisingly displayed a return to a pre-apoptosis state and the disappearance of all apoptotic signs. This phenomenon, called anastasis, refutes the idea that the release of apoptotic executioners from permeabilized mitochondria and following caspase activation are the «death sentence» for the cell [5,17]. Indeed, the same cells treated with sub-lethal doses of BH3-mimetic ABT-737 did not die, although such treatment led to low but detectable levels of MOMP [18]. This became possible since a number of mitochondria in apoptotic cancer cells could remain intact for several reasons. Firstly, upon caspase-mediated cleavage of Bid in cytosol and movement of tBid towards mitochondria, Bax failed to insert and activate in a part of mitochondria and Bak did not oligomerize in the outer membrane of mitochondria that have not undergone MOMP. Secondly, increased levels of Bcl-2/Bcl-XL can protect specific mitochondria from MOMP. Thirdly, the Bcl-2 antagonist ABT-737 induces the permeabilization of intact mitochondria in cells displaying incomplete MOMP (iMOMP) [19]. In fact, in several experimental systems, iMOMP was detected when few mitochondria evade MOMP and then re-populate in the cell [20]. As only a limited number of mitochondria undergo MOMP, the amount of released cytochrome *c* is insufficient to trigger apoptosis, but adequate for sub-lethal caspase activation and the consequent activation of endonuclease, leading to genome instability [18].

Why do some mitochondria resist MOMP, while the others do not? To answer this question, it might be helpful to pay attention to the fact that there is diversity in the mitochondria population within a cell. Mitochondria are the powerhouse of a cell but have a plethora of other vital functions as well, including the regulation of cell death. The number of mitochondria per cell ranges from a few to thousands depending on the type of cells and their energy demands. In some cells, mitochondria exist as single, randomly dispersed organelles, while mitochondria form a dynamic network in other cells that can change the shape and subcellular distribution [21]. For example, in cardiomyocytes mitochondria form clusters and surround the nuclei, thus generating ATP to maintain efficient nucleocytoplasmic communication [22]. In melanocytes, a fraction of mitochondria is in direct contact with melanosomes and these mitochondria-melanosome connections are thought to be relevant for melanocyte biogenesis [21]. However, the heterogeneity of mitochondria is not only found in different types of cells, but also within a single cell. Mitochondria localize in different regions of the cytoplasm within a cell, which could imply dissimilar morphology and functional properties of this organelle. Using image techniques, the heterogeneity of mitochondrial redox potentials, Ca^2+^ levels, statics, and dynamics has been reported for various types of cells [23]. Structural organization of the cell, local demands for ATP, and other cellular needs (e.g., Ca^2+^-buffering capacities) may determine region-specific functional behaviors [24]. Theoretically, it could mean that different intracellular mitochondrial subpopulations have different capacities to undergo MOMP and are thus characterized by the heterogeneity of the survival ability of this organelle.

The possible reasons for mitochondrial heterogeneity include the local environment of mitochondria, different quantities of mitochondrial DNA, epigenetic regulation, and post-translational modifications of mitochondrial gene-encoded proteins [25]. The cristae organization is individual for each mitochondrion and can be modulated in response to altered metabolic demands such as starvation, causing cristae to become narrow, or death signaling, leading to the disorganization of cristae structures. Cardiolipin is an important phospholipid that orchestrates apoptosis by integrating signals from a variety of death-inducing proteins (e.g., tBid and caspase-8) [26,27,28], and also seems to play a crucial role in the maintenance of cristae morphology [29,30]. Cardiolipin comprises approximately 20% of the mitochondrial inner membrane, but there could be fluctuations in the amount of cardiolipin within the cell mitochondria population, which could affect the number of cristae along with other membrane components.

**Figure 1:**
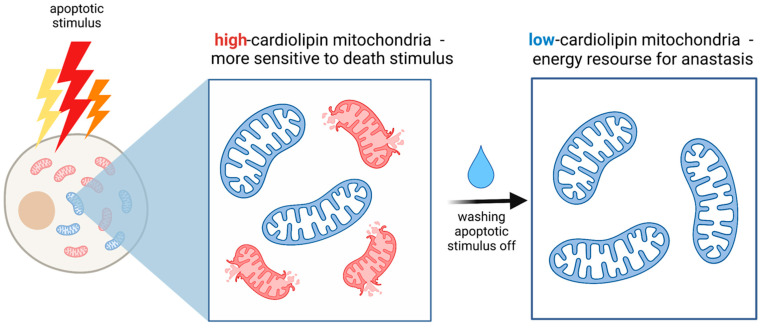
The possible role of mitochondrial heterogeneity in anastasis. The low cardiolipin-contained mitochondria are more resistant to death signals rather than high cardiolipin-contained mitochondria and can therefore be a source of energy for anastasis.

Cardiolipin deficiency results in an increase in mitochondrial size and the absence or disorganization of cristae structures, which are important for apoptosis to proceed. Mitochondria with a low cardiolipin content have low membrane potential, while mitochondria with a high cardiolipin content exhibit higher membrane potential [31]. It was hypothesized that high cardiolipin-containing mitochondria had a large negative charge inside, which would theoretically more attract Ca^2+^ than the less polarized mitochondria, therefore, making them more sensitive to apoptotic stimulus [31]. Thus, low cardiolipin-containing mitochondria can resist death signals and be a source of energy for anastasis afterward (Figure 1).

Another factor regulating a mitochondrial response to apoptotic stimuli could be the mitochondria and endoplasmic reticulum (ER) contacts, which are thought to be a way to transfer Ca^2+^ between these two organelles [32,33,34]. It allows high local concentrations of Ca^2+^ to be created at sites of the ER-mitochondria contacts and the function of Ca^2+^-dependent proteins to be activated. High concentrations of Ca^2+^ in mitochondria can open the mitochondrial permeability transition pore (MPTP), leading to cytochrome *c* release and the consequent activation of caspase cascade [35]. Mitochondria in different cell regions probably have different numbers of contact sites with the ER, which could be another reason why some mitochondria show persistent MOMP and serve as a source of energy during anastasis.

Furthermore, the involvement of ER in anastasis could be not limited by the ER-mitochondria contacts. as ER stress plays a huge role in cell death regulation. At the very early stages of ER stress, the physical coupling between ER and mitochondria is increased, facilitating Ca^2+^ transfer from ER to mitochondria [36]. On the one hand, the increased Ca^2+^ uptake by mitochondria enhances energy substrates production for the cellular adaptive response [37]. On the other hand, if ER-stress persists, Ca^2+^ can activate apoptosis. In addition to Ca^2+^ regulation, chronic or overwhelming ER stress promotes *CHOP* (DNA damage-inducible transcript 3, also known as C/EBP homologous protein) transcription which subsequently can induce apoptosis [38]. It was hypothesized that under persistent stress, cancer cells undergo CHOP-induced apoptosis, which allows them to gain genetic alterations, but once the apoptotic stimulus is removed, cells inhibit the CHOP-pathway (probably via miRNA-211) and survive, becoming even more carcinogenic and metastatic due to an increased mutation number [39]. This hypothesis, based on the ER stress response, could be one of the possible explanations of switching a cell decision from dying to survival path.

Taken together, the diversity of mitochondrial lipid compositions (especially, cardiolipin contents), mitochondrial morphology (e.g., cristae number), and the ER-mitochondria interactions could determine the differences in the responses of mitochondria to stressors within a cell. Intact mitochondria seem to be a key factor determining the ability of cells to undergo anastasis since recovery from death should be a highly energy-consuming process. It could be that the more mitochondrial diversity a cell has, the higher the possibility that some of the mitochondria would provide a way for anastasis.

## 4. Role of Transcription and Post-Transcriptional Modifications in Anastasis

Anastasis is basically the successful overcoming of injury after apoptotic stimulus. However, the idea of limiting the permanent damage in response to temporary stress is not unique for anastasis alone. To a certain extent, the wound healing process aims the same. In both cases, the activation of gene transcription plays a pivotal role. Once stress is eliminated, the cell obtains a possibility to counteract apoptotic processes. It was demonstrated that after washing out ethanol, new RNA synthesis is immediately initiated and the recovery process can be effectively prevented by the transcription inhibitor actinomycin D, indicating that transcription is essential for anastasis [40]. The gene expression changes caused by ethanol treatment were estimated by whole RNA sequencing in mouse primary liver and human liver cancer HepG2 cells. Whole RNA sequencing allowed the time-course of anastasis to be divided into early and late stages. The early stage is characterized by the up-regulated expression of genes involved in the regulation of transcription, proliferation, migration, cell death, and cell survival. During the first 4 h of recovery, genes regulating the TGFβ pathway were particularly up-regulated [40]. It is known that TGFβ acts as a tumor promoter and induces tumor cell migration and epithelial-mesenchymal transition (EMT). Moreover, TGFβ-regulated EMT requires the expression of EMT transcription factors (TFs) such as Snail, Slug, and Twist, which is mediated by phosphorylation and activation of the downstream-located transcription factors Smad2 and Smad3 [41,42]. Indeed, phosphorylated levels of Smad2 and Smad3 were increased within the first 4 h of recovery from apoptosis and then diminished [17]. The Smad3/Smad4 complex binds directly to regulatory promoter sequences of Snail, inducing its transcription, and then Smad3/Smad4 and Snail protein form a transcriptional repressor complex, which can bind to regulatory promoter sequences of *E-cadherin* and *occludin*, thus repressing their transcription [43,44]. Snail acts as an inductor of EMT, for the acquisition of cancer stem cells (CSC)-like traits and chemoresistance [44,45,46]. The knock-down of Snail significantly suppresses recovery after ethanol and staurosporine treatment. It is associated with enhanced caspase activity and Poly(ADP-ribose)polymerase (PARP) cleavage, thus preventing anastasis [17].

The activity of transcription factors, including Snail, can be controlled not only by changing their transcriptional level but also by changing their intracellular localization [47]. To regulate gene expression, Snail must translocate to the nucleus. The import of Snail is mediated by importin β through nuclear localization sequence recognition, and the export is exclusively occurring by Exportin 1 (XPO1/CRM1) through nuclear exclusion sequence recognition. It is well known that nuclear exporters, especially XPO1, are often aberrantly overexpressed in many cancers [48]. Specifically, in anastatic cells, the expression of key molecules in nuclear-cytoplasmic transport (such as XPO1 and importin NUTF2) was highly induced [49]. The inhibition of XPO1 by Selinexor, an exportin inhibitor, leads to Snail nuclear degradation mediated by the nuclear retention of X-box protein FBXL5, which results in reversing the mesenchymal phenotype, growth inhibition, and apoptosis induction [50]. Moreover, the inhibition of XPO1 by Leptomycin B, as well as by shRNA, causes a reduction in the survival of HeLa and MDA-MB-231 cells after paclitaxel and etoposide treatments. Leptomycin B is also significantly reduced acquired chemo-resistance and the invasive capacity of anastatic cells [49]. Whether or not it is linked to Snail degradation is still unclear.

In contrast to TGFβ and Snail pathways, which activated transiently, some angiogenesis-related genes are up-regulated permanently during both early and late stages of anastasis [17]. Among them are two top up-regulated genes throughout recovery, namely, *placenta growth factor (PGF)*, which binds the VEGF receptor, stimulates endothelial cell proliferation and migration [51], and *Ephrin/Ephrin receptor*, which is also important for blood vessel development and angiogenesis [52]. As mentioned above, recovery from cell death requires an increased level of energy supply, and the up-regulation of angiogenesis-related factors aims to provide the nutrition needs and removal of metabolic waste products. At the same time, intense angiogenesis, being a hallmark of cancer, would contribute to cancer cell recovery and is possibly important for tumor recurrence during an interval between anti-cancer treatments.

At the late stage (6 h of recovery), cells tend to switch from proliferation to migration [17]. During this stage, the transcriptional enrichment of a group of genes involved in focal adhesion and cytoskeleton reorganization has been determined [17]. The up-regulation of the *Mmp9*, *10*, and *13* genes [40], which encode metalloproteases, also indicates the intention of anastatic cells to separate from the extracellular matrix, directly contributing to increased cell mobility and migration [53]. By using scratch assay, Sun et al. showed that anastatic cells close the wound faster than untreated cells and a higher number of anastatic cells is characterized by elongated shape at the migration destination compared to mock-treated cells [17]. As the migratory capacity is an essential trait for cells to become invasive [54], the anastatic cells can be more prone to metastasis.

The list of top down-regulated genes during the late stage of anastasis, surprisingly, includes a significant number of genes encoding various histones [40]. It was demonstrated that the degradation of histones in response to DNA damage and consequent chromatin decompaction facilitate the search for homologous sequences and enhance the access of damaged and template DNA, thus contributing to DNA repair. The artificial shutdown of *H3* and *H4* histones in yeast via controlling transcription of these genes by *GAL1–10* promoter also significantly increased chromatin mobility [55]. It is possible that after removing the apoptotic stimulus, histone down-regulation leads to chromatin de-condensation and an attempt to repair damaged DNA. However, chromatin mobility could be a double-edged sword as its misregulation could promote unwanted DNA changes, such as mutations, chromosomal rearrangements and translocations, and chromatin reorganization, which might drive tumorigenesis [55,56]. This chromatin mobility dualism fits the observation that cells acquire numerous DNA alterations after recovery.

The transcriptional profile of apoptotic cells showed similarity with early response during anastasis. These up-regulated genes during both apoptosis and anastasis might be a driving force of apoptosis and the intention to complete it. Alternatively, the products of these genes might counteract apoptosis and try to rescue the cell until their «last sigh». In contrast, cells embarked on the apoptosis pathway accumulate RNAs that encode survival proteins and start translating them only after apoptotic stress is eliminated. If the stress is too strong, mRNAs might degrade and anastasis cannot be initiated. It seems that cells are always poised for the immediate start of recovery by synthesizing survival proteins; however, they just «wait» for the moment the stress is gone [17]. Some of the early anastatic genes are transcribed during apoptosis; for example, *Gadd45g*, which is involved in DNA repair and the cell cycle control. Under apoptotic stress, *Gadd45g* preserves the DNA of dying cells and promotes the repair of damaged cells after stress elimination [40]. Another example is *Rnu6* which encodes U6 small nuclear RNA and plays an important role in the splicing of a mammalian pre-mRNA. The up-regulation of *Rnu6* might be a sign that post-transcriptional regulation is activated in anastasis. Moreover, a switch in post-transcriptional regulation toward allowing translation could be the heart of the matter of anastasis. Besides the accumulation of mRNAs coding for anti-apoptotic and survival factors, which are not translated until the apoptotic stimulus is gone, the possible involvement of post-transcriptional regulation is illustrated by returning some key apoptosis proteins to the pre-treatment level. After apoptosis induction, cells appropriately demonstrated processing of caspase-3 and cleavage of its substrate, PARP; however, after eliminating the death stimulus, cells showed restored levels of full forms of these proteins [17,57]. The interesting fact is that mRNA levels of these proteins are not significantly increased during and after anastasis, allowing us to suggest that not only is the activation of transcription happening during anastasis, but some post-transcriptional changes are crucial for recovery as well.

**Figure 2:**
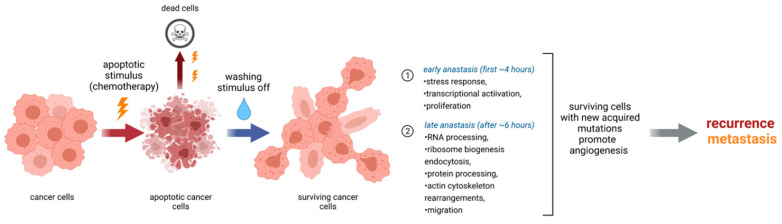
Summary of key events in cells during anastasis. Anastasis can be divided into early and late stages at which different molecular processes dominate. Eventually, cells after anastasis acquire new mutations which make them more prone to recurrence and metastasis after chemotherapy.

On top of that, there are some indications concerning the role of epigenetic regulation in this process. It was shown that cancer cells recovering from apoptosis acquire higher tumorigenicity and metastatic potential in vivo and that anastasis induced the formation of new cancer stem cells (CSCs), which originate from the non-CSCs (NCSCs) in breast cancer cells after staurosporine and paclitaxel treatments [58]. The percentage of CSCs (CD44+/CD24−) in the growing anastatic cell population is significantly higher than in the non-treated population; also, recovered cells display an elevated level of *CD44*, a prominent stemness marker. Importantly, the methylation status of *CD44* and *CD24* determined the expression levels of these genes. In non-treated cells, *CD44* was hypermethylated and *CD24* was hypomethylated, while the hypomethylation of *CD44* and hypermethylation of *CD24* were observed in cells having undergone apoptosis reversal. The inhibition of DNA methylation or demethylation before apoptosis induction results in the reduction of the CSC formation rate after apoptosis reversal. It indicates that epigenetic modifications of CSC marker genes are at least partially responsible for anastasis. It also showed that cells recovering from apoptosis become more prone to CSC-like traits, EMT, and possess enhanced tumorigenicity and metastatic properties in vivo [58] (Figure 2).

Thus, anastasis involves the expression of genes that promote proliferation, survival, migration, and angiogenesis. Activation of transcription is a central process during anastasis; however, post-transcriptional, post-translational (will be discussed below) and epigenetic regulations seem to be important as well. mRNAs of many pro-survival factors are accumulated during apoptosis. Cells have a way back until the very end, but they need a death stimulus to be gone (Figure 2). Therefore, it is intriguing to investigate and understand which mechanisms allow mRNA of survival proteins to be translated.

## 5. Double Role of Caspases

Caspase activation following MOMP is a known hallmark of mitochondria-mediated apoptosis. Accumulated evidence also suggests that caspase activation does not always lead to apoptosis. The same caspases which orchestrate apoptosis are involved in various normal cellular processes such as cell proliferation and the cell cycle [59], regulation of the immune response [60] and neuronal activity [61], learning and memory [62], suppression of necroptotic cell death [63] and others. Some cells, like neurons, can tolerate sublethal caspase activation without triggering apoptosis for days [64], indicating that activated caspase cascade alone is not the «point-of-no-return» [65].

Caspase-3 is a major player in the execution phase of both extrinsic and intrinsic apoptosis pathways mediating downstream terminal effects of cell demolition. Caspase-3 involvement in tumor repopulation after cancer therapy and in promoting genome instability and tumorigenesis has been demonstrated [66]. Heavily irradiated mouse breast cancer cells stimulated the growth of non-irradiated cancer cells when seeded together [66]. To determine whether caspase-3 is responsible for this effect, the comparison of caspase-3-deficient and -proficient cells was performed and found that the latter were significantly more effective in promoting the growth of cancer cells in vitro and the growth of tumors in vivo. Based on this observation, a model where a key role of prostaglandin E2 (PgE2), which is known to promote tumor growth and stem cell proliferation, in tumor repopulation was suggested [67,68]. According to this model, the caspase-3 downstream signaling pathway involves phospholipase A2 (PLA2) activation leading to the production of PgE2. Indeed, the production of PgE2 in *casp3−/−* MEF cells and 4T1shcasp3 cells after irradiation was considerably reduced compared to wild-type, whereas transduction of a constitutively active Pla2g6 (a gene which encodes a function equivalent protein to the caspase-cleaved version of iPLA2) substantially restored PgE2 production in *casp3−/−* MEFs [66]. It has also been shown that sub-lethal doses of radiation increase the ability of caspase-3 to facilitate carcinogenesis [12]. In fact, such «sub-lethal caspase-3 activation» promotes permanent double-strand DNA breaks, the frequent emergence of chromosomal abnormalities, and acquire the capacity of anchorage-independent growth, which is a hallmark of malignant transformation [69]. This also indicates the role of caspase-3 in causing and sustaining genomic instability in response to DNA damage. The moderate doses of radiation of MCF10A human mammary cells cause cytochrome *c* release and persistent caspase-3 activation while having normal morphology with no signs of apoptosis [12]. Although, as mentioned above, caspase-3 is considered as the main executor in apoptosis, its activity does not lead to cell death necessarily and depends on the amount of its active form. It is possible that a sub-lethal amount of active caspase-3, which might appear under iMOMP in anastatic cells, also contributes to malignancy. Interestingly, melanoma cells in presence of sub-lethal caspase activation gained increased migration and invasion rates in vitro and in vivo [70]. However, the pan-caspase inhibitor Q-VD-OPh prevented neither the migration nor invasion of survived cells, indicating that active caspases do not play an essential role in acquiring these properties or that caspases might provoke the migration program and then become dispensable.

At the late stage (6 h) of anastasis, several of the cell cycle arrest genes display the peak of transcription [40]. One of them is *Cdkn1a* that encodes p21, the cyclin-dependent kinase inhibitor [71]. Upon genotoxic stress *p53* transcriptionally up-regulates *p21*, which acts as a promoter of DNA repair and growth arrest. However, depending on cellular localization, nuclear or cytoplasmic, p21 could act as either a tumor suppressor or a tumor promoter, respectively [72]. Cytoplasmic p21 could lead to mitotic slippage after p21-induced growth arrest in tumor cells, stimulate transcription of secreted factors with mitogenic and antiapoptotic activities and abrogate apoptosis [73]. Cytoplasmic p21 directly interacts with procaspase-3 upon DNA damage and prevents procaspase-3 processing via the formation of the procaspase 3-p21 complex [74]. In addition, p21 was shown to form a complex p21/Cdk4/PCNA that induces the cell cycle entry leading to cell survival [75]. Human hepatoma HepG2 cells were resistant to Fas-mediated apoptosis because of procaspase-3/p21 complex formation and the direct inhibition of activated caspase-3 by XIAP [75]. Furthermore, the induction of p21 is correlated with resistance to TGFβ-mediated apoptosis [76]. At the same time, p21 was suggested to function as an inhibitor of various DNA repair pathways (nucleotide excision repair, base excision repair, mismatch repair), which require PCNA (proliferating cell nuclear antigen) by disrupting PCNA interaction with DNA repair molecules as well as promoting PCNA degradation [76]. Paradoxically, p21 itself could be cleaved by caspase-3 and the 15 kDa cleavage product of p21 could facilitate caspase-3-directed apoptosis [77]. Apparently, the localization of p21 and the balance between its full and cleaved forms are involved in the cell fate decision. The up-regulation of Cdkn1a during anastasis could contribute to recovery by preventing caspase-3 activation and inducing the cell cycle entry with DNA damage. In addition, during anastasis the induction of nuclear export, as discussed above, could lead to the accumulation of p21 in the cytoplasm, thus making it act as a tumor promoter. Recovered cells exhibit a high number of DNA alterations and at this point, an increased level of p21 could favor malignancy by inhibiting some of DNA repair mechanisms.

Caspase activity is regulated at the transcriptional and post-translational levels. Two forms of caspase-3 can be generated by alternative splicing: a full-length caspase and a short form missing exon six, which results in its opposite effects. Thus, a full-length form drives cells to apoptosis while a truncated one antagonizes it. An increase in the expression ratio of caspase-3s/caspase-3 was shown to associate with chemoresistance of breast carcinoma [78]. While the overexpression of procaspase-3 strongly increased the ability of MCF-7 cells, normally deficient in caspase-3, to undergo apoptosis in response to etoposide and methotrexate, expression of caspase-3s did not sensitize the cells to drug-induced apoptosis. Moreover, the co-expression of both procaspase-3 and caspase-3s totally suppressed the ability of procaspase-3 to sensitize cells to etoposide and methotrexate-induced death. The structure of caspase-3s predicts no catalytic activity due to an absence of the cleavage site between the small and large subunits, the cysteine residue, and sites for binding caspase inhibitors such as XIAP [79]. Caspase-3s failed to bind the apoptosome, therefore, it might be considered an antagonist of apoptosis [79]. It is known that some tumors are associated with the prevalence of one protein isoform over the others due to a defective splicing system [80,81]. There are two possible explanations of how caspase-3s and anastasis could be connected. Firstly, the presence of caspase-3s could determine the ability of cells to undergo anastasis and, thus, expression of caspase-3s could be a predictive marker for malignancy. Secondly, after apoptotic stress elimination, a switch from the long caspase isoform to a short one might take place. If so, it would be interesting to know the mechanisms of this splicing shift and its potential targets during cancer therapy. Thus, caspase-3 might play not only a key role in apoptosis but also in anastasis. The possibility of the cell to choose the recovery pathway could depend on the amount of cleaved caspase-3, the interaction of procaspase-3 with p21, and the splicing shift toward the short caspase-3 isoform. It is important to note that not only human and mouse cells can survive in presence of active caspase-3. Many years ago, it was found that an evolutionarily conserved homolog of caspase-3, the core programmed cell death executioner protein CED-3, promotes the initiation of neuronal regeneration in *Caenorhabditis elegans* [82]. Moreover, active CED-3 is present in the embryonic neurosecretory motor neuron (NSM) in neuroblasts, which divide to give rise to the larger NSM. As a consequence, these NSMs survive and differentiate into serotonergic motor neurons [83]. Finally, CED-3 might function in asymmetric cell division and cellular polarization in lineages other than cell death lineages, which might be a crucial event for normal animal development [84].

## 6. A Role of Nuclear Changes in Anastasis

Regardless of the pathway triggering apoptosis, the culmination of apoptosis is DNA fragmentation, which is also a hallmark and a critical step of this type of cell death [85]. However, in certain situations, it seems to not be the point-of-no-return. The release of EndoG and apoptosis-inducing factor (AIF) from the mitochondria during MOMP causes chromatin condensation and lesions in DNA structure [86]. The cells acquire numerous mutations, which they are not able to repair immediately and completely because the repair enzymes, including PARP, are already cleaved by caspases or inhibited. This is one of the main reasons why MOMP is thought to uncompromisingly lead to cell death, and as already discussed, if the stress stimulus is removed, apoptosis is interrupted.

During anastasis, it takes some time before a new portion of pro-surviving proteins is synthesized and initiates the restoration of DNA alterations. RNA sequencing of HeLa cells treated with ethanol demonstrated the up-regulation of genes that counteract pro-apoptotic factors [17]. For example, the chaperone HSP70 could retain AIF in the cytoplasm and suppress AIF activity, thus preventing further DNA damage [40]. The cell cycle arrest genes, such as *btg1*, *cdkn1a*, and *trp53inp1*, were also activated indicating possible DNA repair. The expression of PARP and ICAD returned to the pre-apoptosis level, disabling the inhibition of DNA repair and caspase-activated DNase (CAD) activation, respectively. Restored DNA repair in anastatic cells was demonstrated using the comet assay for single- and double-strand DNA breaks [5]. It showed prominent comet tails from cells before washing ethanol off, and the comet tails disappeared from most liver and NIH 3T3 cells after its removal [5], indicating that the broken DNA was repaired, although the quality of repair is questionable.

After washing off the apoptotic stimulus, most of the cells exhibited normal nuclei but at the same time, there was an increase in the number of cells with micronuclei compared to untreated cells [5,40]. The emergence of micronuclei is well-known evidence of acquiring chromosomal abnormalities and mitotic catastrophe. Micronuclei formation occurs during the first cell division after the reversal of apoptosis and even during the division of the daughter cells. The karyotyping of metaphase-arrested cells 3 days after the induction and removal of apoptotic signals showed a significant increase in chromosomal aberrations such as variations in chromosome number and mis-joining of broken chromatids. A possible result of genetic alterations is the promotion of the phenotypic diversity of cancer cells [87]. Indeed, a fraction of anastatic cells exhibited loss of contact growth inhibition and anchorage-independent growth [5], which are the signs of transformed and malignant phenotypes [88]. Thus, it also supports the notion that anastasis might be carcinogenic.

However, besides being a marker of chromosomal abnormalities, micronuclei seem to have another function—they could contribute to DNA repair in the nucleus. Consequently, it can be involved in DNA repair after apoptotic damage thus allowing cells to survive. Homologous recombination (HR) is considered to be a key pathway of DNA repair in somatic mammalian cells, where the central role belongs to the Rad51 protein [89]. It was shown that after radiation-induced DNA damage the number of cells with focally concentrated protein Rad51 was significantly increased among TGR-1 fibroblasts compared with non-treated cells [90]. Although the amount of protein Rad51 was not changed, its distribution within the cell altered significantly. Shortly after irradiation, Rad51 foci were distributed evenly throughout the nuclei. At 16 h after treatment, they formed clusters and linear strings and Rad51 foci were extruded from the main nuclei into micronuclei after 2 days. The formation of the single strand (ss) DNA-Rad51 filaments are thought to mediate homologous DNA recognition and initiate an exchange between ssDNA and homologous double-strand DNA [91]. DNA repair by HR is typically enhanced in TP53-deficient polyploid tumor cells and is considered an antagonistic force to apoptosis [92], but it can still be compatible with simultaneous chromatin sorting by autophagy in the same cells [93]. Autophagy of micronuclei was demonstrated in the human osteosarcoma U2O3 cell line, where autophagic micronuclei exhibited a reduced chromatin content as compared to non-autophagic micronuclei. The former contained damaged DNA and were surrounded by damaged envelopes, suggesting chromatin degradation by lysosomal enzymes. In this case, autophagy via the elimination of micronuclei contributes to surviving and maintaining genome stability [94].

**Figure 3:**
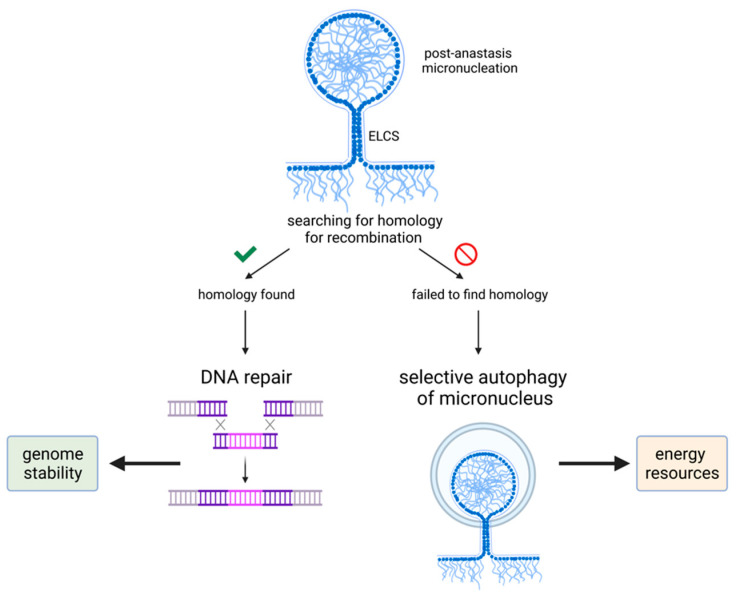
Micronucleus fate in surviving cells. Post-anastasis cells show the increased formation of micronuclei, which is a marker of DNA damage. However, micronuclei could contribute to maintaining genomic integrity by providing DNA material for homologous recombination or be used as a resource of energy in case of a failure to find sequence homology in micronuclei.

After genotoxic treatment, several cell lines were characterized by endopolyploidization and exhibited autolysosomes at the nucleo-cytoplasmic border, which were positive for the markers of autophagic vacuoles and the lysosomal activation (cathepsin B) [93]. The chromatin inside the autolysosomes was selectively degraded, suggesting that chromatin is sorted into two categories, namely, preservation and autodigestion. If chromatin is too damaged to be repaired by HR, Rad51 loses its proper association with Rad52 and appears in autolysosomal vacuoles as clusters of linearly arranged polymeric fibers. The chromatin in these vacuoles is destined to be degraded via selective autophagy [93]. Thus, genomically unstable endopolyploid tumor cells (ETCs) could use chromatin autophagy to get rid of excessive and/or irreparable genetic material that may favor its survival after genotoxic treatment (Figure 3). Similarly, since micronuclei can represent a post-effect of anastasis, surviving cells could induce chromatin sorting as well to eliminate defective chromatin and increase genome stability after recovery (Figure 3).

Both autophagic and non-autophagic micronuclei were in close vicinity to the main nuclei of polyploid cells or formed chromatin buds, allowing us to suggest that they could be connected with the nuclei through the nuclear envelope [95]. There are particularly notable structures called envelope limited chromatin sheets (ELCS), which are characterized by extensions of the nuclear envelope that surrounds one or more ~30-nm-thick chromatin sheets [96]. ELCS is thought to be a result of aberrant mitosis or mitotic slippage after genotoxic treatment or spindle perturbation and is used as a marker of poor prognosis in leukemia [97]. The cells where ELCS appeared after failed mitosis did not undergo immediate cell death. They eventually died, but via extremely protracted and delayed apoptosis. These observations might suggest that the appearance of ELCS precedes the point-of-no-return of cell death; however, mitotic catastrophe cannot be excluded [97]. As a possible mechanism of delaying apoptosis via ELCS, it was suggested that micronuclei could provide DNA material for HR and deliver it to the main nucleus by ELCS through nuclear rotation [94]. If the repair system fails to find a homologous site, the micronuclei are fated for selective autophagy [95]. Therefore, both non-autophagic and autophagic micronuclei may contribute to facilitating genome stability in ETCs. This model could explain why the majority of micronuclei are not observed in the process of autophagic degradation. They can be instead engaged in DNA repair and chromatin resorting [95]. Besides micronuclei, in cytoplasmic pockets of ELCS mitochondria and multiple autophagic vesicles are often observed in genotoxic-treated endopolyploid lymphoma cells [97]. It could mean that the autophagy of cytoplasmic contents cooperates with autophagic removal of chromatin in micronuclei for maintaining genome stability by at least two mechanisms: (1) by constructing DNA material for cells undergoing protracted DNA repair, and (2) more directly, by providing nutrients and consequently the energy needed for nuclear rotation by microtubules at the site of the ELCS pockets [95]. Taken together, it became clear that micronuclei could provide an effective road to anastasis.

## 7. Anastasis and Other Forms of Cell Death

As pointed out above, anastasis was described as a mechanism of survival, which is opposed to apoptosis. At present, the Nomenclature Committee on Cell Death has recognized more than ten different forms of cell suicides, among which in addition to apoptosis are autophagy, necroptosis, ferroptosis, pyroptosis, and others [98]. A link between apoptosis and anastasis was discussed extensively; however, a possible crosstalk between anastasis and other cell death forms is less established. We have mentioned the role of autophagy in nuclear changes essential for anastasis. Energy supply in the form of amino acids and fatty acids should be especially important for anastatic cells to keep recovering since their mitochondria are damaged after failed apoptosis and unable to support cells with energy properly. It was shown that after 6 h of recovery from apoptotic stimuli, some autophagy-related genes (*Atg12*, *Sqstm1*) were up-regulated [40]. It is reasonable to suggest that after severe stress, recovery would require the removal of damaged DNA and organelles, especially permeabilized mitochondria, which are not compatible with normal metabolism. However, recovery from apoptosis and recovery from starvation via autophagy seems to be two different processes based on differences in the activity of the up-regulated genes [17]. Moreover, anastatic cells display elongated morphology, an increased level of migration and wound healing, while cells recovered from autophagy had the same rate of wound closure as mock-treated cells [17]. These differences once again indicate that anastasis and recovery via autophagy after starvation are distinct processes.

Nevertheless, there are few reasons to consider autophagy being actively involved in anastasis. Cell recovery and return to life imply the elimination of all damaged cellular components and waste products. The canonical autophagy pathway also referred to as macroautophagy, is terminated in lysosomes, leading to degradation of the cargo. Autophagy is also implicated in the unconventional secretory pathway, where autophagosomes do not maturate and instead are secreted from cells [99,100]. It could be an alternative way for the clearance of damaged or toxic materials during anastasis. The autophagy-mediated secreted factors may stimulate cellular proliferation via auto- and paracrine signaling and promote an invasive phenotype [101]. EMT is associated with autophagy, which helps cells to come through stressful environmental and intrinsic conditions [102]. As anastatic cells are also prone to EMT, it creates a possible link between autophagy and anastasis. In fact, this link is an interesting perspective to study a connection between autophagy with micronuclei and ELCS as well.

It was recently shown that several types of cells can reverse and survive after necroptosis in a process termed resuscitation [103]. Survival following necroptosis has been observed upon chemical inactivation of two main necroptotic proteins, RIPK3 and MLKL. This process is regulated by the endosomal sorting complexes required for transport-III (ESCRT-III)-mediated membrane repair, which prevents plasma membrane disruption during necroptosis [103]. As mentioned above, during the crosstalk between apoptosis and anastasis several growth-regulated genes are activated. Similarly, the survival of necroptotic cells was associated with an up-regulation of growth factors, highlighting mitogenic signaling during this type of cell recovery [104].

Various microbial infections, as well as several endogenous damage-associated molecules, can activate pyroptosis. The cleavage of gasdermin results in the formation of specific pores in the plasma membrane, which is essential for pyroptosis execution [105]. It was suggested that ESCRT can function to repair gasdermin pores, leading to the reversal of pyroptosis. As pyroptosis is associated with the production of cytokines such as IL-1β and IL-18 [106], the ESCRT repair pathways may help cells to prolong their inflammatory function by delaying death.

Resistance to anoikis, another form of cell death, is a critical step for tumor cell invasion [2]. Whether this resistance is associated with anastasis is still not clear; however, the up-regulation of the epidermal growth factor receptor (EGFR) family plays an important role in overriding anoikis. Moreover, as stated above, during EMT, the loss of E-cadherin, activation of TGF-β signaling via the transcription factors Twist and Snail, and N-cadherin expression are associated with anastasis and can lead to anoikis resistance followed by increased tumor invasiveness.

Ferroptosis is a non-apoptotic iron-dependent form of programmed cell death defined by the requirement for iron and an accumulation of cellular ROS for this cell death execution [98]. As compared with apoptosis reversal, the removal of ferroptosis inducers, such as erastin and glutamate, does not help ferroptotic cells to survive [107]. However, with the removal of cell death inducers simultaneously with the addition of the reduced form of glutathione or the radical-trapping antioxidant ferrostatin-1, ferroptotic cells can be rescued and recovered [107]. It is important to note that, although various inhibitors of ferroptosis can prevent this form of cell death, none of them can reverse the initiated process, suggesting regulatory distinctions between preventing and reversing ferroptosis [108].

Thus, altogether, these results reveal evidence that other types of cell death might also be reversible and can lead to the discovery of new strategies to diminish their reversibility, as well as developing new models for studying the physiological, pathological, and therapeutic potential of cell recovery processes.

## 8. Detection of Anastasis

Apoptosis reversal was reported as morphological features before and after the treatment of growing cells with various apoptotic stimuli; however, it could not provide strong evidence of back from the brink [5]. Therefore, the real-time monitoring of caspase activity would be more demonstrative. The detection of activated caspases as a sign of apoptosis or for its own sake is a routine procedure, while the detection of anastasis faces some technical challenges [109,110]. The cells recovered from apoptosis appear morphologically almost indistinguishable from surrounding healthy cells and a biomarker of anastasis has not been identified yet [111]. There are several developed biosensors, which allow survival after caspase activation and anastasis to be confirmed not only in cultured cells but in vivo as well [111]. For instance, the dual-color CaspaseTracker bio-sensor system allowed anastasis to be detected during *Drosophila melanogaster* development [110]. It fluoresces red in cells with current or recent caspase activity and constantly fluoresces green in cells that have experienced caspase activity in the past, with or without ongoing caspase activity.

Apart from cell visualization, the most widely used methods for the detection of cell death are western blotting, flow cytometry, and the viability assay (methods based on as-saying of ongoing cellular metabolism and enzyme activity, such as MTT) which reflects summarized and averaged response of a large number of cells. A significant disadvantage of the cell population-based analysis is obscuring the presence of functionally important subpopulations in tumors. While a high percentage of cancer cells die after cytotoxic treatment, cells undergoing polyploidization, senescence, fusion, and anastasis could be neglected in most preclinical assays [112,113]. For example, endopolyploid tumor cells can be mistakenly scored as dead and also do not form macroscopic colonies during the time period (approx. 10 days) of the standard clonogenic assay. However, their presence should be taken into consideration since these giant cells are generated by clinically relevant doses of chemotherapy and could be a result of mitotic catastrophe. Moreover, these giant cells are fairly viable, can produce tumor-promoting factors, and have the potential to give rise to heavily malignant progeny [112]. Another example is that detecting cleaved caspase-3, cleaved PARP or other pro-apoptotic factors (release of cytochrome *c*, Bax, and Bak oligomerization) is usually sufficient to confirm apoptosis, but these factors can all be detected during anastasis as well [5,17,40]. This consideration makes the recognition of standard apoptotic signs in cell population-based assays not reliable enough to answer the question of whether a specific treatment was truly successful. At this point, the live single-cell analysis is the most precise way to determine the real efficacy of anti-cancer treatment and allow the prediction of relapse because of surviving subpopulations. Unfortunately, single-cell assays are more complicated methods compared to cell-based population assays and not very affordable, which discourages their widespread use as preclinical tests for the evaluation of treatment cytotoxicity. Therefore, as cells recovered from death are more aggressive and genomically unstable, it is extremely important to distinguish dead from dying cells.

## 9. Conclusions

The identification of the anastasis phenomenon and the accumulated data on it indicate the importance of developing a new direction in the study of tumor resistance. Although the phenotype of anastatic cells is characterized in detail, there is a lack of new experimental data to shed light on the mechanisms behind recovery from apoptotic stimulus. Recovery from the other forms of cell death (necroptosis, pyroptosis, ferroptosis, autophagy) has been studied even less. The most comprehensively described component of anastasis is changed in gene transcription; however, it seems to be a consequence and not a cause. Anastasis challenges cancer treatment as it not only allows cells to survive, but also favors their further malignancy. On the contrary, anastasis could be beneficial for the treatment of neurodegenerative and cardiological disorders as it prevents the undesirable death of non-dividing cells.

Unfortunately, we have still not come close to the underlying root of why cells can reverse death. Is it a common ability for all types of cells or do only some of them tend to anastasis? Does it arise from genetic heterogeneity or the differences in the microenvironment? Is there a marker to predict propensity for anastasis? What determines which ’not-apoptosis’ route the cells should go: anastasis, senescence, or mitotic catastrophe? In addition, it is most important to know how anastasis can be prevented in vivo. Once we can shed light on these questions, it will bring us one great step closer to successful cancer treatment. Thus, further research into unique data sets will undoubtedly reveal new information about the mechanisms that control each of these processes and how they might be exploited therapeutically.
